# Sulforaphanes: disruptors of phagophores and autolysosomes

**DOI:** 10.1080/27694127.2022.2067642

**Published:** 2022-05-01

**Authors:** Yan Zhou, Wei Wu

**Affiliations:** a Department of Biochemistry and Molecular Biology, School of Basic Medical Sciences, Capital Medical University, Beijing 100069, China; b Beijing Key Laboratory for Tumor Invasion and Metastasis, Capital Medical University, Beijing, China

**Keywords:** Apoptosis, microtubule, mitophagy, proteostasis, sulforaphane

## Abstract

Sulforaphane and its metabolites (SFNs) cause apoptosis in cancers and could be potential anti-cancer drugs. We focused on investigating the underlying mechanisms through which SFNs inhibit cancers. First, SFNs cause microtubule disruption by phosphorylated MAPK1/ERK2-MAPK3/ERK1-mediated activation of 26S proteasome leading to a microtubule-associated protein degradation and microtubule depolymerization. Second, SFNs cause the accumulation of autophagosomes and mitophagosomes via blocking their fusion with lysosomes. These changes might be involved in multiple signaling pathways. High-performance liquid chromatography-tandem mass spectrometry showed that SFN regulates the expression of lipoproteins; highly expressed FASN (fatty acid synthase) correlates with cancer malignancy and poor prognosis. More, SFN lowers the expressions of FASN, ACACA (acetyl-CoA carboxylase alpha), and ACLY (ATP citrate lyase) by activating the 26S proteasome; SFN inhibits the interactions of TUBA/α-tubulin with FASN, ACACA or ACLY; SFN also reduces the production of intracellular fatty acids; knockdown of FASN increases mitochondrial abnormality and apoptosis. Moreover, SFN decreases the expressions of mitophagy-associated proteins BNIP3L/NIX and BNIP3 and the interaction between BNIP3L/NIX and LC3-II/-I and upregulates mitochondria-associated LC3-II/-I. Therefore, SFN might cause apoptosis via inhibiting the microtubule-mediated lipoprotein activity and the fusion of lysosomes with autophagosomes and mitophagosomes.

Using TUBA/α-tubulin to normalize loading in western blot analysis, we surprisingly noticed that SFNs remarkably degrade this house-keeping protein by activating the 26S proteasome. Further, we discover that SFNs disrupt microtubules by degradation of STMN1 (stathmin 1), and depolymerization of TUBA/α-tubulin and TUBB/β-tubulin heterodimers. As feedback in response to SFNs, the levels of HSPA/Hsp70 and autophagy marker protein MAP1LC3/LC3 (microtubule associated protein 1 light chain 3)-II are upregulated in whole-cell and mitochondrial lysates, while autophagosomes and mitophagosomes are accumulated as highlighted by transmission electron microscopy [1]. We reveal that LC3-II links autophagosomes to lysosomes by LAMP1 (lysosomal associated membrane protein 1). Combined with bafilomycin A_1_, a lysosomal inhibitor, SFNs block autophagosome- and mitophagosome-lysosome fusion by decreasing the colocalization of LAMP1 with LC3-II causing the accumulation of autophagosomes and mitophagosomes. Eventually, SFNs-triggered dysfunction of microtubules, and disturbance of autophagy and mitophagy causes apoptosis.

SFNs downregulate nearly 350 proteins as assessed via high performance liquid chromatography-mass spectrometry. These proteins cover, but are not limited to, components of the cytoskeleton, oncoproteins, cell cycle proteins, mitochondrial membrane translocases, RNA polymerases, and metabolic enzymes. Moreover, SFNs upregulate the 26S proteasome via sustained MAPK1/ERK2-MAPK3/ERK1 axis activation by phosphorylation, leading to microtubule disruption and apoptosis. The degradation of TUBA/α-tubulin and TUBB/β-tubulin in the cytosol could be 26S proteasome- dependent. Co-immunoprecipitation shows that TUBA/α-tubulin probably interacts with proteins such as HSPA/Hsp70, FASN, ACACA, ACLY and PFKFB4 (6-phosphofructo-2-kinase/fructose-2,6-bisphosphatase 4)^1^, which may primarily form aggregated complexes to function specifically in vivo. No 26S proteasome in mitochondria is detected, but CASP3 (caspase 3) is verified to cleave mitochondria-bound TUBA/α-tubulin. SFNs activate mitochondrial CASP3 causing TUBA/α-tubulin cleavage, and the depolymerization of TUBA/α-tubulin-TUBB/β-tubulin dimers. Tissue microarray shows that increased TUBA/α-tubulin contributes to cancer malignancy. Thus, degradation or cleavage of TUBA/α-tubulin might cause cytoskeletal disassembly, increase the formation of apoptotic bodies, and inhibit cell cycle progression and cell motility.

Aberrant mitochondria are seen in the SFNs-treated non-small cell lung cancer (NSCLC) cells, and the reduction of mitochondrial membrane potential is determined. SFNs-induced protein damage and mitophagy interference might result in mitochondrial swelling. Some cytosolic protein molecules, such as TIGAR (TP53 induced glycolysis regulatory phosphatase) might translocate into mitochondria. Both TUBA/α-tubulin and LC3-II are thought to be the vehicles for transport of these cytosolic proteins and also play scaffold roles for protein conformation stabilization and signaling transduction. Consequently, disequilibrium of microtubule dynamics might initiate the signaling networks from cytosol to mitochondria. Cytosolic HSPA/Hsp70 gives a quick response to SFNs to maintain protein conformation or transport misfolded proteins into mitochondria for degradation.

Protein degradation influences proteostasis through the 26S proteasome, CASP3 and lysosomal proteolytic enzymes in the cytosol and mitochondria; over-accumulated misfolded proteins might result in the reduction of mitochondrial membrane potential, mitochondrial swelling and mitochondria permeabilization. Mitophagy helps cells to degrade or remove misfolded proteins to maintain mitochondrial proteostasis to control whole-cell protein quality, conformation, and activity. An ongoing project in our laboratory is to verify that SFNs upregulate HSPA/Hsp70 and HSPA9/mHsp70 and promote its interaction with TUBA/α-tubulin or LC3-II in the cytosol and on mitochondria. Also, SFNs promote the binding of TUBA/α-tubulin to LC3-II, while lower interactions of TUBA/α-tubulin to TOMM20, TIMM23, and TIMM17A. The degradation for misfolded proteins in mitophagy could be reduced by SFNs-induced inhibition of autolysosome formation.

FASN is involved in the de novo synthesis of fatty acid (FA) and lipids in tumor cell. Studies show that the abnormality of membrane lipids might trigger membrane fluidity disruption and interfere with the fusion of two membrane-bound organelles, such as autophagosomes and lysosomes. FASN is highly expressed in tumors and is positively associated with malignant grades of NSCLC by immunohistochemical staining in microarray tissues. Also, the survival of NSCLC patients is negatively related to FASN level. Therefore, it is interesting to establish a new anti-cancer therapy via targeting FA synthesis. Here, we report that SFNs decrease FASN and its binding to microtubules resulting in mitochondrial phospholipid reduction and apoptosis. Simultaneously, SFNs also downregulate ACACA, ACLY and transcription factor SREBF1/SREBP1 by the 26S proteasome. Further, SFNs lower the interactions of TUBA/α-tubulin with FASN, ACACA, or ACLY. Furthermore, SFNs reduce the yield of intracellular FA and mitochondrial phospholipids; knockdown of FASN decreases mitochondrial membrane potential, causes deformation in cell membrane structure and mitochondrial morphology, and increases reactive oxygen species leading to apoptosis. These indicate that FASN is a promoter for cell proliferation, which might influence mitochondrial structures and functions.

It is reported that FASN inhibitors increase the accumulation of LC3-II in a couple of cancers. The phospholipid in the mitochondrial membrane is mainly cardiolipin, located in the mitochondrial inner membrane, and it is transferred to the outer membrane when the mitochondria is damaged, triggering mitophagy. Our results show that the total free FA and mitochondrial phospholipids are reversely increased after treatment with CCCP. This suggests that SFNs decrease mitochondrial phospholipid levels, thereby disrupting the mitochondrial membrane. Both BNIP3L/NIX and BNIP3 localize to the outer mitochondrial membrane by way of a C-terminal transmembrane domain. Also, those two proteins directly interact with the phagophore membrane protein LC3-II/-I. BNIP3L/NIX recruits LC3-II/-I to envelope damaged mitochondria. Here, we find that SFNs downregulate mitophagy regulators BNIP3 and BNIP3L/NIX, upregulate mitochondrial-associated LC3-II/-I, and inhibit the interaction between BNIP3L/NIX and LC3-II/-I. These results demonstrate that SFN might inhibit the formation of phagophores; besides, SFNs inhibit the fusion of autophagosomes or mitophagosomes with lysosomes causing the accumulation of the former. Interestingly, knockdown of TUBA/α-tubulin downregulates BNIP3L and BNIP3 production, and upregulates LC3-II/-I. Further, SFNs reduce the interaction and colocalization of TUBA/α-tubulin with BNIP3L. Thus, SFNs might cause apoptosis via inhibiting FA production and microtubule-mediated mitophagy.

The working concentration (15 µM) in cell culture limits SFNs with regard to their use for clinical trial. However, we demonstrate that combination with other anti-cancer agents (such as paclitaxel) will lower the effective concentration of SFNs to 2 µM which patients might tolerate. In addition, development of new SFNs via modifying the chemical groups of this molecule or establishment of nano-SFN molecules might increase the working efficiency and lower the effective concentration. In fact, we also uncover that PFKFB4, one of the enzymes of glycolysis, is downregulated in NSCLC and brain glioma cells. PFKFB4 is highly expressed in many tumors and its levels are correlated with cancer malignancy. SFNs are not only anti-cancer candidates, but also might be potential glucose controllers. The investigation of SFN-induced cell signaling (Figure 1) will help us understand cell motility, growth, trafficking, autophagy, and death to design more powerful drugs to treat diseases.

**Figure 1.**
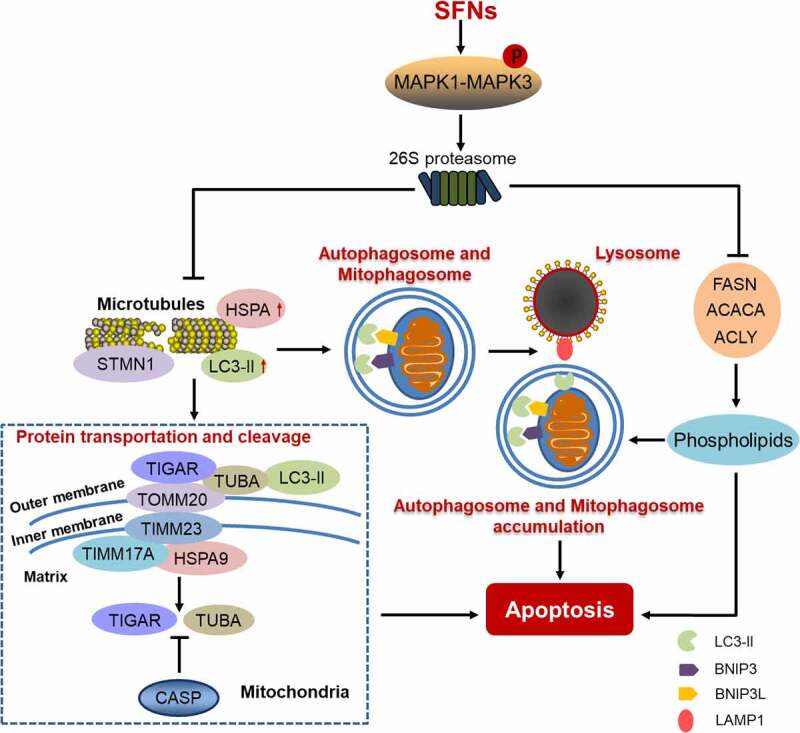
A proposed schematic of the signal pathways whereby SFN causes apoptosis via targeting FA synthesis and inhibiting mitophagy in NSCLC cells.

## Data sharing statements

No additional data are available.

## References

[1] Yan Y, Zhou Y, Li J, et al. Sulforaphane downregulated fatty acid synthase and inhibited microtubule-mediated mitophagy leading to apoptosis. Cell Death Dis. 2021; 12(10):917. PMID: 34620841. 34620841 10.1038/s41419-021-04198-2 PMC8497537

